# Cognitive and emotional-volitional disorders in patients with residual schizophrenia

**DOI:** 10.1192/j.eurpsy.2023.1056

**Published:** 2023-07-19

**Authors:** V. Mitikhin, D. Oshevsky, L. Alieva

**Affiliations:** Department of Mental Health Support Systems Research Centre, Mental Health Research Centre, Москва, Russian Federation

## Abstract

**Introduction:**

Studies show that patients diagnosed with residual schizophrenia are characterized by cognitive and emotional-volitional disorders that increase with age. They can be the main barrier to treatment, psychosocial rehabilitation, and cause disability.

**Objectives:**

To identify cognitive and emotional-volitional disorders in patients with residual schizophrenia.

**Methods:**

The BACS and the abbreviated MMPI test were used; 20 patients with residual schizophrenia (ICD-10 F.20.5xx) receiving outpatient treatment (mean age 59.65±14.24 years) were examined. Exclusion criterion: scores more 4 on at least one parameter of positive symptoms according to the PANSS.

**Results:**

Patients with residual schizophrenia show an overall decline in cognitive function (BACS composite score = 31.56±14.24) compared with healthy individuals, as well as compared with patients suffering from other forms of schizophrenia spectrum disorders. The greatest deficiency was revealed in the speed of information processing (subtests “Symbol Coding”=28.01±10.06; “Verbal Fluency”=37.56±11.57) and auditory-speech memory (subtest “Verbal Memory”=33.25±6 .02). These parameters showed significant associations (r=0.56 at p≤0.01) with the disability of such patients. However, this deficit could be compensated by the relative preservation of planning processes and executive functioning (subtest “Tower of London” = 14.91±4.57). Among the emotional and volitional disorders, the most important is the subjective feeling of low mood and paranoid tendencies (MMPI scales “Dp” = 56.38±10.74T, “Pa” = 59.06±14.49T), which can reduce the compliance of patients with residual schizophrenia.

**Conclusions:**

Methods for leveling cognitive and emotional-volitional disorders should to include in programs of psychosocial rehabilitation of patients with residual schizophrenia.

**Disclosure of Interest:**

None Declared

